# Characterisation of and risk factors for extended-spectrum β-lactamase producing *Enterobacterales* (ESBL-E) in an equine hospital with a special reference to an outbreak caused by *Klebsiella pneumoniae* ST307:CTX-M-1

**DOI:** 10.1186/s13028-022-00621-6

**Published:** 2022-02-09

**Authors:** Katariina Thomson, Katarina Eskola, Marjut Eklund, Kristiina Suominen, Merita Määttä, Jouni Junnila, Suvi Nykäsenoja, Kati Niinistö, Thomas Grönthal, Merja Rantala

**Affiliations:** 1 Department of Equine and Small Animal Medicine, Faculty of Veterinary Medicine, University of Helsinki, PO Box 57, 00014 Helsinki, Finland; 2 Department of Veterinary Biosciences, Faculty of Veterinary Medicine, University of Helsinki, PO Box 66, 00014 Helsinki, Finland; 3 Oy 4Pharma Ltd, Arkadiankatu 7, 00100 Helsinki, Finland; 4 Finnish Food Authority, PO Box 100, 00027 Helsinki, Finland; 5 Veterinary Teaching Hospital, Faculty of Veterinary Medicine, University of Helsinki, PO Box 57, 00014 Helsinki, Finland

**Keywords:** Bacterial infection, Cluster, Horse, Multi-drug resistant bacteria, Nosocomial infection, Surveillance

## Abstract

**Background:**

Extended-spectrum β-lactamase producing *Enterobacterales* (ESBL-E) are important causative agents for infections in humans and animals. At the Equine Veterinary Teaching Hospital of the University of Helsinki, the first infections caused by ESBL-E were observed at the end of 2011 leading to enhanced infection surveillance. Contact patients were screened for ESBL-E by culturing infection sites and rectal screening. This study was focused on describing the epidemiology and microbiological characteristics of ESBL-E from equine patients of the EVTH during 2011–2014, and analysing putative risk factors for being positive for ESBL-E during an outbreak of *Klebsiella pneumoniae* ST307.

**Results:**

The number of ESBL-E isolations increased through 2012–2013 culminating in an outbreak of multi-drug resistant *K. pneumoniae* ST307:*bla*_CTX-M-1_:*bla*_TEM_:*bla*_SHV_ during 04–08/2013. During 10/2011–05/2014, altogether 139 ESBL-E isolates were found from 96 horses. Of these, 26 were from infection-site specimens and 113 from rectal-screening swabs. A total of 118 ESBL-E isolates from horses were available for further study, the most numerous being *K. pneumoniae* (n = 44), *Escherichia coli* (n = 31) and *Enterobacter cloacae* (n = 31). Hospital environmental specimens (N = 47) yielded six isolates of ESBL-E. Two identical *E. cloacae* isolates originating from an operating theatre and a recovery room had identical or highly similar PFGE fingerprint profiles as five horse isolates. In the multivariable analysis, mare–foal pairs (OR 4.71, 95% CI 1.57–14.19, P = 0.006), length of hospitalisation (OR 1.62, 95% CI 1.28–2.06, P < 0.001) and passing of a nasogastric tube (OR 2.86, 95% CI 1.03–7.95, P = 0.044) were associated with being positive for ESBL-E during the *K. pneumoniae* outbreak.

**Conclusions:**

The occurrence of an outbreak caused by a pathogenic ESBL-producing *K. pneumoniae* ST307 strain highlights the importance of epidemiological surveillance of ESBL-E in veterinary hospitals. Limiting the length of hospitalisation for equine patients may reduce the risk of spread of ESBL-E. It is also important to acknowledge the importance of nasogastric tubing as a potential source of acquiring ESBL-E. As ESBL-E were also found in stomach drench pumps used with nasogastric tubes, veterinary practices should pay close attention to appropriate equipment cleaning procedures and disinfection practices.

**Supplementary Information:**

The online version contains supplementary material available at 10.1186/s13028-022-00621-6.

## Background

Multi-drug resistant bacteria are one of the biggest threats for human and animal health. It has been estimated that by year 2050 antimicrobial resistance will annually cause more human deaths than cancer if the development of resistance continues to accelerate [1]. Extended-spectrum betalactamase-producing *Enterobacterales* (ESBL-E) and especially ESBL *Klebsiella* spp. are important causative agents of difficult-to-treat nosocomial infections in both humans and animals, and the high occurrence of ESBL-E is a worldwide challenge [2–4]. Infections caused by ESBL-E are especially troublesome in equine medicine, as only a limited number of antimicrobial agents are suitable for horses.

Similar ESBL-E strains and ESBL enzymes have been isolated from humans and animals and the detection of these can indicate possible transmission through contact with animals as well as food [2]. As humans are in close contact with horses kept as companion animals, the risk for transmission of bacterial strains between horses and humans cannot be neglected. Drug-resistant *Enterobacterales* among horses have been assumed to be acquired from the environment or from people [5]. The effectiveness of the transmission of ESBL-characteristics is based on mobile genetic elements, and resistance genes can move from one bacterial species to another [6]. For example, dozens of plasmids have been identified that often carry multiple antimicrobial resistance genes in various *K. pneumoniae* isolates [7]. The vast research interest in antimicrobial resistance in animals has so far been in livestock, however companion animals and horses have been somewhat overlooked. There have been few reports on the nosocomial spread of ESBL-E in small animal and equine hospitals [8, 9].

This study describes the characteristics of ESBL-E in equine patients at a veterinary teaching hospital in Finland, with a special reference to an outbreak of *K. pneumoniae* ST307:CTX-M-1. This strain of *K. pneumoniae* is known to have caused outbreaks in human hospitals, e.g. a neonatal intensive care unit [10, 11], and occurrence and transmission of this strain have also been reported in small animal veterinary practices [12, 13]. However, no outbreak of ESBL -*K. pneumoniae* ST307 has been reported in horses previously. The study will also shed light on some possible risk factors for horses being positive for ESBL-E in a hospital setting. This information will aid in understanding the importance of control measures in the prevention of antimicrobial resistant pathogens in equine clinical settings.

Sporadic ESBL-E isolates have been observed in companion animal and equine infections in Finland since 2004 [14]. In 2011, the Clinical Microbiology Laboratory (CML) of the Faculty of Veterinary Medicine, serving the Equine Veterinary Teaching Hospital (EVTH) of the University of Helsinki, reported several subsequent equine ESBL-E infections at EVTH, followed by an increase in frequency in 2012. In 2013, an outbreak of *K. pneumoniae* was identified as multiple subsequent discoveries of a similar isolate of *K. pneumoniae* (based on antibiogram and phenotypic ESBL production) were recognized from bacterial cultures. Other ESBL-E species were also observed at the same time.

The aims of this study were: (1) to describe the epidemiology and microbiological characteristics of ESBL-E isolates derived from horses at the EVTH during 2011–2014; and (2) to analyse putative risk factors for ESBL-E infection or gut carriage during an outbreak of *K. pneumoniae* in a veterinary equine hospital.

## Methods

### Setting and source population

The EVTH of the University of Helsinki is the only veterinary teaching hospital in Finland and admits both primary and referral equine patients. The case load is approximately 2600 horses annually. Primary cases arrive from the greater Helsinki area and referral cases are accepted from the whole country. According to the EVTH patient record data, a mean of 70 foals is treated at the hospital during spring and summertime and most stay in the hospital for some days. The EVTH can house up to 24 single horses or mare–foal pairs, however, all stalls are seldom occupied simultaneously. There is an isolation unit with three stalls, but horses can also be cohorted in groups of five where each cohort has its own entrance and anteroom for separation of clean and contaminated areas. Approximately 220 surgeries are performed at the EVTH on an annual basis. The surgeries consist of emergency procedures, such as colic surgeries, and elective procedures, such as castrations and arthroscopies.

The EVTH has a hospital hygiene program including a surveillance system for nosocomial infections and multidrug resistant bacteria. If a horse has a clinical infection, an infection site specimen is always obtained. According to the hygiene policy, an ESBL-E finding in a hospitalised patient initiates contact patient tracing and screening for ESBL-E carriage (rectal swab). This is performed in order to recognize a possible outbreak and to observe whether standard hygiene precautions are effective (i.e., no spread of the ESBL-E of concern among hospitalised horses). As the first ESBL-E positive specimen is indicative of transmission, usually only one positive specimen was obtained per horse. However, in some cases, as infections sites are always cultured, a positive rectal swab may have preceded an ESBL-E positive infection site specimen. If the horse, however, had an ESBL-E positive infection site, a rectal swab was not routinely obtained afterwards. For descriptive epidemiology, we wanted to describe the clinical infections due to clinical relevance whereas for the risk factor study, the data for potential risk factors were only collected from the point of admission up until the first ESBL-E positive specimen.

The source population of this study consisted of the equine patients hospitalised ≥ 24 h at the EVTH during October 2011–May 2014. All adult horses were housed in separate stalls with no possibility for direct contact with other patients. Mares and suckling foals were housed together in the same stall.

### Bacteriological specimens

Infection site specimens were taken aseptically with a cotton swab (M40 Copan Diagnostics, Italy) or by aspiration from abscesses (Portagerm®, bioMérieux, France) by the treating veterinarian. Screening specimens were taken from the rectum of the horse and from possible infection sites (if any) with a cotton swab (M40). Environmental specimens were taken with a sterile swab or cotton gauze from stomach drench pumps, nasogastric tubes, the operating room, and recovery room premises (operation table, top of the anaesthesia unit, enterotomy lavage hose, floor, doorstep, and soft padding of a drain).

### Microbiological methods

Culture of clinical and environmental specimens and species identification were performed as described by Garcia and Isenberg [15]. Bacterial species were also identified by matrix-assisted laser desorption/ionization time-of-flight (MALDI-TOF) mass spectrometry (Bruker MALDI Biotyper Microflex LT, Bruker Daltonik GmbH, Bremen, Germany). Susceptibility testing was executed according to CLSI guidelines [16]. The disc diffusion test was performed for the following antimicrobial agents: amikacin, gentamicin, amoxicillin/clavulanic acid, cefpodoxime, sulphamethoxazole/trimethoprim, enrofloxacin, and doxycycline (Oxoid Ltd., UK). Phenotypic identification of ESBL-E was performed using the double-disc diffusion test [17], and MASTDISCS® Combi (Mast Group, UK) according to the manufacturer’s instructions. In addition, the susceptibility to colistin (Colistin ETEST®, bioMérieux, France) was tested for selected *Enterobacter cloacae*, *Escherichia coli*, *K. pneumoniae*, *Citrobacter* spp., *Enterobacter aerogenes* and *Klebsiella oxytoca* isolates that represented each PFGE-clone, including PFGE subclusters. If no veterinary-specific susceptibility breakpoints were available in the aforementioned standards, human CLSI breakpoints were used [18].

### Descriptive epidemiology

To detect temporal clustering of different ESBL-E strains, all ESBL-isolates (n = 139) collected during October 2011–May 2014 were plotted by month and by bacterial species in a histogram. To determine the number of horses (n = 96) with an ESBL-E and the source of ESBL-isolates (i.e., infection or colonisation/asymptomatic carrier), laboratory and patient data were combined from the respective information systems (Provet Net, Finnish Net Solutions, Finland) of the CML and the EVTH.

### Analytical epidemiology and statistical analyses

The risk factor study concerned a five-month outbreak period of ESBL *K. pneumoniae* from April 2013 to August 2013, during which time many other ESBL-E were also detected. An ESBL case was defined as a horse that had been treated at the EVTH ≥ 24 h during the aforementioned period, and had returned an ESBL-E positive result from an infection site and/or from a screening specimen after ≥ 24 h of hospitalisation. A control was a horse from the same population but had been ESBL-E negative in screening and/or infection-site specimens. Outpatients and patients with any ESBL-E finding were excluded from the control group. Data for potential risk factors for being positive for ESBL-E were collected for each case during their hospitalisation from the point of admission up until the first ESBL-E positive specimen (cases) or the latest date of an ESBL-E negative specimen (controls). Each horse was considered as an independent individual, however, mare and foal housed together also formed a separate variable.

Data on basic demographics and potential risk factor variables of the cases (n = 52) and controls (n = 90) were collected in a Patient Data Collection Form (Additional file 1) using Epi-Info (Epi-Info v. 7, CDC, USA). The potential risk factors for being positive for ESBL-E were first assessed using univariable logistic regression models, after which a stepwise multivariable logistic regression analysis was conducted for the risk factors with a P-value < 0.05 in the univariable analyses. In the stepwise selection process, a significance level of 0.15 was required to allow a variable into the multivariable model, and a significance level of 0.20 was required for a variable to stay in the multivariable model. Only the main effects of the factors were studied (i.e. the analysis did not include any interactions between risk factors). For sensitivity purposes, a penalized LASSO (least absolute shrinkage and selection operator) logistic regression model [19, 20] was fitted. In the LASSO-modelling, Akaike Information Criteria (AIC) was used as the criteria for the optimal model selection and the Nesteroy’s optimization as the optimization technique. Odds ratios (OR) with 95% confidence intervals (CI) were calculated. P-values (Wald) < 0.05 were considered statistically significant. All statistical analyses were performed using SAS System for Windows, version 9.4 (SAS Institute Inc., USA).

### Pulsed-field gel electrophoresis (PFGE)

Of the total of 139 ESBL-E isolates, 118 isolates from 84 horses were available for further investigation. PFGE typing was performed for the 118 isolates according to the PulseNet O157 protocol [21]. Clonal similarity of the strains was determined [22] and analysed by UPGMA-cluster analysis with 0.5% optimization and 1% Dice band matching tolerance with a ≥ 85% similarity cut off value using GelCompar II software version 6.5 (Applied Maths NV, Belgium).

### Multilocus sequence typing (MLST)

MLST typing was performed for selected *E. coli* (n = 28) and *K. pneumoniae* (n = 7) isolates that represented each PFGE-clone, including subclones. For *E. coli*, the MLST was performed as described by Grönthal et al. [23]. For the MLST of *K. pneumoniae* we used allele-specific primers [24] that were attached to the universal sequencing primers [25] as described by the Pasteur Institute, Protocol 2 [26]. The *K. pneumoniae* MLST PCR reaction mixture (20 µL in total) contained 10 µL of 2 × Phusion Flash High Fidelity Master Mix (Thermo Fisher Scientific, Waltham, Massachusetts, USA), 0.25 µM of each primer, and 1 µL of DNA template. The PCRs were run in three separate protocols: the *rpoB* products were amplified as follows: initial denaturation at 98 °C for 15 s; 30 cycles of denaturation at 98 °C for 2 s, annealing at 52 °C for 10 s, and elongation at 72 °C for 15 s; final elongation at 72 °C for 1 min. The running conditions for the remaining genes were the same, except the annealing temperature was 67 °C (*gap*A*, inf*B, *mdh*) or 62 °C (*pho*E*, ton, pgi*).

### PCR and sequencing for ESBL-gene families

The 118 isolates available for further study were investigated for the carriage of the cefotaxime-hydrolysing ESBL (CTX-M), Temoneira β-lactamase (TEM), and sulfhydryl variant ESBL (SHV) genes by multiplex-PCR [23]. In addition, grouping was performed by sequencing for CTX-M-1, -M-2, -M-8, -M-9 or -M-25 for isolates that represented each PFGE-cluster or subclusters as follows: *E. coli* (n = 14), *K. pneumoniae* (n = 8), *Citrobacter* spp. (n = 1), and *K. oxytoca* (n = 2) [23]. All sequence analysis was carried out using CLC Main Workbench (v. 7).

## Results

### ESBL-E isolations and isolation sites

**Fig. 1:**
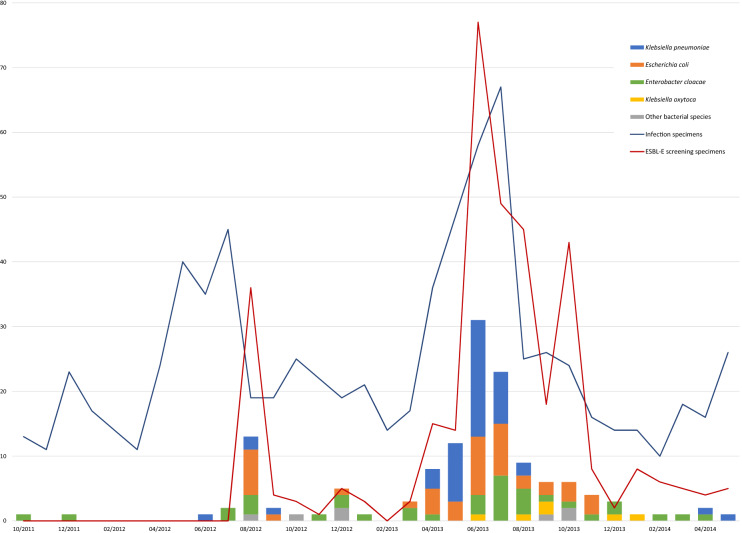
Occurrence of ESBL-E isolates in rectal screening specimens and infection specimens during the study period. The number of obtained specimens (lines) and found ESBL-E isolates (columns) are presented on the y-axis, and the timeline by month and year on the x-axis

Altogether, 785 infection site specimens from 584 horses and 354 screening specimens from 274 horses were studied for ESBL-E isolates during October 2011–May 2014 at the CML. Of these, ESBL-E was found in 25/785 (3%) infection site specimens and 84/354 (24%) screening specimens. There were 13 horses which had both an ESBL-E positive infection site specimen and a positive rectal swab. The 25 infection site specimens yielded 26 isolates of ESBL-E, and the 84 screening specimens yielded 113 isolates. Altogether, there were 139 ESBL-E isolates found during October 2011 to May 2014 (Fig. 1). Sixty percent of these ESBL-E isolates (n = 83) were found during the five-month outbreak, from April to August 2013. Figure 1 shows a histogram by time and species including the monthly number of investigated specimens indicating sampling activity.

**Fig. 2:**
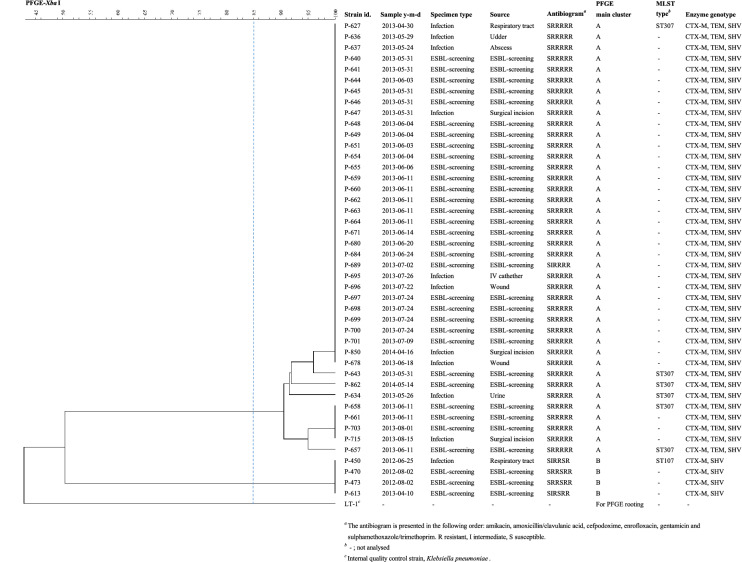
PFGE clustering of the 44 *Klebsiella pneumoniae* isolates from horses

The first ESBL-E isolate, *E. cloacae,* originated from a surgical site infection after a colic surgery of a 16-year-old Finnhorse gelding in late 2011. The second one, *K. pneumoniae*, was isolated from a clinical respiratory tract specimen of a 4-day old warmblood filly in June 2012. This finding was the first case of a minor *K. pneumoniae* ST107:*bla*_CTX-M-1_ clone consisting of four cases in June 2012 – April 2013. The most striking increase in the number of ESBL-E occurred during April – August 2013, when a major clone of 38 multi-drug resistant (MDR) *K. pneumoniae* ST307:*bla*_CTX-M-1_ isolates emerged indicating an outbreak. In addition to beta-lactam antibiotics, the members of this clone were resistant to gentamicin, trimethoprim-sulfonamides, enrofloxacin and doxycycline (Table 3, Fig. 2).

Out of all ESBL-E isolates, 118 isolates from 84 horses were available for further investigation. Of these, the most numerous bacterial species was *K. pneumoniae* (n = 44), followed by *E. coli* (n = 31), *Enterobacter cloacae* (n = 31), *K. oxytoca* (n = 6), *Citrobacter* spp. (n = 4), and *Enterobacter aerogenes* (n = 2). Table 1 shows the sites of origin of the ESBL-E isolates included in the microbiological study. The majority of horses (56/84; 67%) that were positive for ESBL-E had only one ESBL-E isolate, while 22 horses (26%) had two, and six horses (7%) had three ESBL-E isolates.

**Table 1:** Description of ESBL-E isolates available for further study, obtained from horses during October 2011 – May 2014

Bacterial species	Specimen type		Infection site (N = 25)							
Isolates N = 118 (%)	Rectal screening^a^ N = 93 (%)	Infection^b^ N = 25 (%)	Abscess	Blood	IV catheter	Udder	Respiratory tract	Surgical incision	Wound	Urine
*K. pneumoniae* n = 44 (37)	33 (35)	11 (44)	1	–	1	1	2	3	2	1
*E. cloacae* n = 31 (26)	20 (22)	11 (44)	–	–	–	–	–	9	2	–
*E. coli* n = 31 (26)	29 (31)	2 (8)	–	1	–	–	1	–	–	–
*K. oxytoca* n = 6 (5)	6 (6)	–	–	–	–	–	–	–	–	–
*Citrobacter* spp. n = 4 (3)	3 (3)	1 (4)	–	–	–	–	–	1	–	–
*E. aerogenes* n = 2 (2)	2 (2)	–	–	–	–	–	–	–	–	–

^a^Twenty-six horses were asymptomatic carriers of two or three ESBL-E isolates (n = 49)

^b^Eleven horses had a clinical infection caused by two or three ESBL-E isolates (n = 13)

Isolates of different species of extended-spectrum β-lactamase producing *Enterobacterales* (ESBL-E) were obtained from specimens originating from horses at the Equine Veterinary Teaching Hospital (EVTH) during the study period

Environmental specimens (N = 47) investigated during December 2012 – September 2013 also revealed ESBL-E isolates (n = 6). Table 2 presents the sampling locations and the found species.

**Table 2:** ESBL-E isolates from environmental specimens during December 2012 – September 2013 at the EVTH

ESBL-E finding	Surface	Sampling date yyyy-mm-dd	Isolate no
*E. cloacae*	Colic operating theatre: operating table	2012–12–17	P-567
*E. cloacae*	Pooled specimen: from the recovery room of the colic operating theatre: floor and doorstep	2013–04–08	P-615
*E. cloacae*	Pooled specimen: enterotomy lavage hose, top of the anaesthesia unit, floor of the recovery room	2013–04–04	P-616
*E. coli*	Pooled specimen: four stomach drench pumps	2013–09–18	P-743
*E. coli*	Pooled specimen: four stomach drench pumps	2013–09–18	P-744
*Citrobacter* spp.	Pooled specimen: from the recovery room of the colic operating theatre: doorstep and soft padding of a floor drain	2012–12–17	P-566

Extended-spectrum β-lactamase producing *Enterobacterales* (ESBL-E) isolates of different species were found from environmental specimens, which were obtained during December 2012–September 2013 at the Equine Veterinary Teaching Hospital (EVTH)

### Results of geno- and phenotypic analyses

During the whole study period 44 K*. pneumoniae* isolates were observed, of which 40 (91%) belonged to the same PFGE cluster with only slight variation in PFGE fingerprints (Table 3, Fig. 2). Of the seven *K. pneumoniae* isolates for which MLST was performed, six were of ST307 and one of ST107 (Fig. 2). Three *K. pneumoniae* isolates with a different PFGE pattern (52% similarity compared to the major clone) were detected before the outbreak (during June–August 2012). The majority (n = 39, 89%) of *K. pneumoniae* isolates clustered temporally within the five-month outbreak of ESBL-*K. pneumoniae* ST307 (April–August 2013), whereas two isolates of the major clone were detected after the outbreak (April – May 2014).

**Table 3:** Geno- and phenotypic data of ESBL-E clusters during October 2011–May 2014

Species	PFGEcluster	No. of isolates	MLST type^*a*^		Antibiogram^*c*^						
				*bla* gene^*b*^	AK	CN	AMC	CPD	SXT	ENR	DO
*K. pneumoniae*	A	40	ST 307	CTX-M-1, SHV, TEM	S	R	I/R	R	R	R	R^*d*^
	B	4	ST 107	CTX-M-1, SHV	S	R	I/R	R	R	S	R
*E. coli*	C	8	ST 167	CTX-M-9, TEM	S	R	S/R	R	R	R	R^*d*^
*E. coli*	D	3	ST 167	CTX-M-1, TEM	S	R	R	R	R	R	R
*E. coli*	E	2	ST 141	SHV, TEM	S	R	I	R	R	S	R
*E. coli*	F^*e*^	2	ST 8107	SHV, TEM	S	R	S	R	R	S	R
*E. coli*	G	2	Unidentified Type	CTX-M-1, TEM	S	R	I	R	R	I	S
*E. cloacae*	H	9	–	SHV, TEM	S	R	R	R	R	R	S/R
*E. cloacae*	I	6	–	SHV, TEM	S/I	R	R	R	R	S	R
*E. cloacae*	J	3	–	SHV, TEM	S	R	R	R	R	I	R
*E. cloacae*	K	2	–	SHV, TEM	S	R	R	R	R	S/R	R
*E. cloacae*	L	2	–	SHV, TEM	S	R	R	R	R	I/R	R
*E. cloacae*	M	2^*e*^	–	SHV, TEM, TEM^*f*^	S	R	R	R	R	S	R
*K. oxytoca*	N	4	–	CTX-M, TEM, SHV	S	R	S	R	R	S	R
*K. oxytoca*	O	2	–	CTX-M, TEM, SHV^*g*^	S	R	S	R	R	S	R
*Citrobacter spp.*	P	2	–	SHV, TEM	S	R	R	R	R	I	R
*E. aerogenes*	Q	2	–	SHV, TEM	S	R	R	R	R	S	R

Geno- and phenotypic description of the extended-spectrum β-lactamase producing *Enterobacterales* (ESBL-E) clusters from the equine and environmental specimens obtained at the Equine Veterinary Teaching Hospital (EVTH) during the study period. Single type representatives are excluded

^*a*^Altogether, 7 representative isolates of *K. pneumoniae* and 28 isolates of *E. coli* were investigated in MLST

^*b*^In addition to multiplex-PCR results, CTX-M gene group was determined by sequencing for representative isolates. TEM = wild-type TEM

^*c*^AK amikacin, CN gentamicin, AMC amoxicillin/clavulanic acid, CPD cefpodoxime, SXT sulphamethoxazole/trimethoprim, ENR enrofloxacin, DO doxycycline, R resistant, I intermediate, S susceptible. Susceptibility annotated with a division sign indicates susceptibilities of different isolates

^*d*^One isolate was sensitive to doxycycline

^*e*^Includes two surface hygienic specimens

^*f*^One isolate carried *bla*_TEM_ only

^*g*^One isolate carried *bla*_SHV_ and *bla*_TEM_ only

**Fig. 3:**
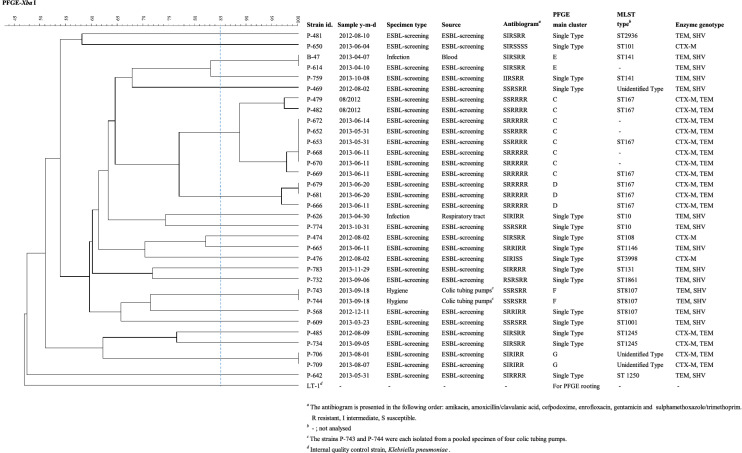
PFGE clustering of the *Escherichia coli* isolates from horses (n = 31) and equine hospital equipment (n = 2)

Among the 31 ESBL-*E. coli* equine isolates, 24 pulsotypes were seen in four clusters of two to eight isolates each (Fig. 3). Sixteen isolates (52%) were singletons. Altogether 26 representatives of the 31 *E. coli* isolates from horses were analysed in MLST, resulting in 13 published *E. coli* sequence types: ST167 (n = 7), ST10 (n = 2), ST141 (n = 2), ST1245 (n = 2), and ten single representatives (Fig. 3). Three isolates were not identified in MLST.

The PFGE profiles of ESBL-*E. coli* isolates from environmental specimens and horses differed. The isolates from stomach drench pumps were of ST8107, as was the closest horse isolate (P-568), however still with less than 72% similarity.

**Fig. 4:**
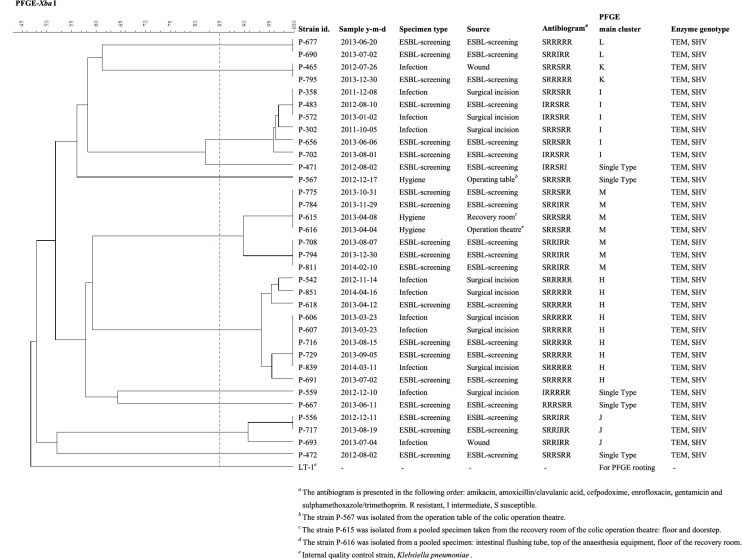
PFGE clustering of the *Enterobacter cloacae* isolates from horses (n = 31), equine hospital surfaces and equipment (n = 3)

Results for the remaining 34 isolates of *E. cloacae*, *K. oxytoca*, *Citrobacter* sp. and *E. aerogenes* are presented in Table 3 and Fig. 4. Apart from *K. oxytoca*, these isolates presented broad heterogeneity.

Of the 118 ESBL-E isolates from horses, nearly all were resistant to gentamicin (116/118 isolates; 98%), trimethoprim-sulfamethoxazole (115/118; 97%), doxycycline (105/118; 89%), and more than half to enrofloxacin (65/118; 55%) (Table 3). Only two isolates (2%) were resistant to amikacin. *Enterobacter* spp. and *Citrobacter* spp. commonly showed both ESBL and AmpC phenotype, whereas isolates of *Klebsiella* spp. and *E. coli* expressed only ESBL phenotype. Fifty-four isolates were tested for colistin and all were susceptible.

### Risk factors for ESBL-E infections

In multivariable analyses, the length of hospitalisation (OR 1.62, 95% CI 1.28–2.06, P < 0.001), being a mare–foal pair (OR 4.71, 95% CI 1.57–14.19 P = 0.006), and undergoing a nasogastric intubation (OR 2.86, 95% CI 1.03–7.95, P = 0.044) were associated with being positive for ESBL-E during the outbreak (Table 4). On average, horses that tested positive for ESBL-E stayed in the hospital for 4.3 days, whereas the average stay of control horses lasted for 1.7 days. The sensitivity analysis confirmed the presented results.

**Table 4:**
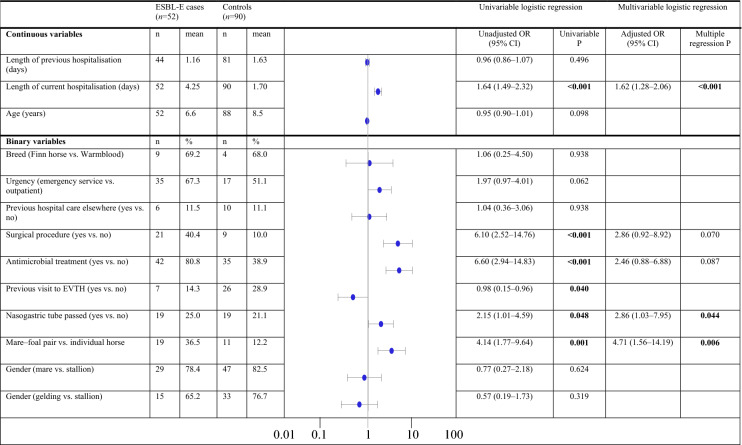
Factors associated with being positive for ESBL-E at the EVTH during the outbreak

Risk factors associated with the acquisition of extended-spectrum β-lactamase producing *Enterobacterales* (ESBL-E) during the *Klebsiella pneumoniae* ST307 outbreak at the Equine Veterinary Teaching Hospital (EVTH) during April **–** August 2013. The number of horses being compared to among cases and controls as well as their proportion is displayed for each variable studied. Risk factors with a P-value < 0.05 are highlighted

## Discussion

Many ESBL-E positive specimens, both from infection sites and rectal swabs, were found in horses at the EVTH between October 2011 and May 2014. Most of the findings were *K. pneumoniae, E. coli*, and *E. cloacae*. During the 31-month study period, a major ESBL *K. pneumoniae* ST307:*bla*_CTX-M-1_ outbreak of five months (April–August 2013) occurred at the EVTH, indicating nosocomial transmission. This sequence type of *K. pneumoniae* has rapidly disseminated globally and has become a significant pathogen in humans [27]; it is known to have caused outbreaks in human hospitals [10, 28]. This strain has also been reported to cause infections in companion animals such as dogs and cats [29, 30]. However, our study is the first to describe an outbreak of ESBL-*K. pneumoniae* ST307:CTX-M-1 in an equine hospital. The occurrence of the outbreak we describe underlines the importance of epidemiological ESBL-E surveillance in veterinary hospitals.

The ESBL-E isolated in our study caused several kinds of infections, most commonly in surgical sites, wounds, and the respiratory tract. The most common species causing infections was *K. pneumoniae* ST307. This is in line with the findings in the study by Shnaiderman-Torban, where *K. pneumoniae* and *E. cloacae* were the most frequently isolated species of ESBL-E from clinical infections in horses during hospitalisation [31]. The antibiograms of the ESBL-E causing infections were very similar to each other regardless of the ESBL-isolate; all of the isolates were resistant to cefpodoxime and trimethoprim/sulphonamides and all, except one, were resistant to gentamicin. Widespread resistance to fluoroquinolones was also noted. Multidrug resistance indicated by resistance to three or more different antimicrobial classes [32] was noted for all ESBL-E isolates that caused a clinical infection in our study population. The resistance to different antimicrobials was also very similar to the antibiograms published in the study by Shnaiderman-Torban [33]. The only remarkable difference was the susceptibility to amikacin; in our study 92% of the strains were susceptible to amikacin, whereas only 76% of the ESBL-E were susceptible to amikacin in the Shnaiderman-Torban study [33]. This might reflect the habits of antimicrobial usage as horses in Finland extremely seldom receive amikacin due to financial restraints whereas in Israel most foals admitted to the hospital receive ampicillin and amikacin for broad-spectrum antimicrobial coverage [34].

Walther et al. [35] revealed a broad heterogeneity of ESBL-producing bacterial species, as was seen in our study. The most common genes associated with ESBL-production in the German study were *bla*_CTX-M-1_ and *bla*_SHV-12_. According to Schmiedel et al. [36], the subtype *bla*_CTX-M-1_ was a common finding in equine ESBL-E isolates. These results are consistent with our findings, where the same genes were found among different species of *Enterobacterales*. This could be explained by horizontal gene transfer. A study by Dolejska et al. [37] found that *E. coli* isolates from horses, environmental smears, and flies at an equine clinic and a riding centre harboured a plasmid, which carried *bla*_CTX-M-1_. In addition, the plasmids contained numerous other resistance genes, which could explain the broad resistance among the different bacterial species.

Among the ESBL *E. coli* isolated in our study, three new and numerous previously identified ST types were found, which reflects the heterogeneity of the population. Similarly, a diverse *E. coli* population was found in a Dutch study describing the occurrence and molecular characteristics of ESBL/AmpC producing *E. coli* in faecal samples from horses in an equine clinic, indicating that clonal nosocomial spread was not the only reason for the high occurrence of *E. coli* [3]*.* Strains positive for *bla*_CTX-M-1_ and *bla*_CTX-M-2_ predominated, and ST10 among others was the most common sequence type [3]. ST10 was also reported by Walther et al. [35] in several equine patients in a German veterinary teaching hospital. However, only two isolates represented ST10 *E. coli* in our study.

The occurrence of ESBL-E in food-producing animals in Finland is very low, but it has not been investigated in healthy horses in Finland [38]. The first confirmed case of ESBL-E in a horse in Finland was in 2004 [14], although it has been found extensively in horses in different parts of the world. In the Netherlands 10.8% of healthy horses [39] and in Turkey 53.5% of healthy racehorses [40] were found to be positive for ESBL-E. A recent report from Israel by Shnaiderman-Torban et al. [31] showed an occurrence of 21% of ESBL-E in horses in farms, with a statistically significant increase in hospitalised horses (77.9% occurrence). *E. coli* and *K. pneumoniae* were frequently isolated in the study. A German study by Walther et al. [35] showed a 10.7% (34/318) incidence of ESBL-E in faeces and open wounds of equine patients, and 94% of these ESBL-E–positive specimens yielded ESBL *E. coli*. ESBL genes and MDR *E. coli* have also been found in riding centres and stable surroundings [5, 41], and the spread of ESBL-producing *Enterobacterales* species has also been shown in other European equine clinics and hospitals, for example in the Czech Republic [37], Germany [8], and the Netherlands [3]. The environment can be a significant reservoir for ESBL-E and resistance genes and potentially allow for transmission to horses [42–44].

From an epidemiological point of view and when attempting to control a hospital outbreak, it is highly relevant to identify all horses that are possible sources for environmental contamination and direct transmission. These include both equine patients suffering from an infection caused by ESBL-E, and asymptomatic horses that shed ESBL-E to the hospital environment in their excrement. Horses excrete large amounts of faeces uncontrollably and thus cause substantial contamination of their environment. It has been shown that horse faeces have been the main vehicle for ESBL infections in equine hospitals [45, 46]. Therefore, it is important to also identify asymptomatic carriers by obtaining rectal screening swabs.

Other sources of contamination, such as the hospital environment and fomites, are also important to investigate as any source might be relevant for a successful control of the outbreak. *Klebsiella* spp. strains have been reported to persist in human hospital environments, regardless of thorough surface cleaning [47]. Moreover, while *Klebsiella* spp. are ubiquitous in nature, transmission can also occur via communal surfaces and fomites [7]. Among human isolates of *K. pneumoniae*, the ST307 genome is reported to encode novel genetic factors, like a plasmid-located gene cluster for glycogen synthesis, and chromosomally-encoded virulence traits including fimbriae [48]. Capsulated ST307 isolates have also shown higher resistance to complement-mediated killing. These features may advantage the strain regarding persistence in the hospital environment and adaptation to the human host [48], but it is unknown whether these factors apply to horses.

Our study revealed several risk factors associated with ESBL-E during the outbreak. The length of hospitalisation was positively associated with ESBL-E carriage. This has also been shown in studies on ESBL-E in humans [49, 50]. In previous studies on horses, staying in an equine hospital has been shown to increase the carriage rate of ESBL-E [31, 34]. Our results indicate that ESBL-positive horses stayed in the equine hospital on average over two days longer than those that tested ESBL-negative. Longer hospitalisation exposes the patient to more handling and procedures by the hospital staff, which may contribute to the transmission of ESBL-E. This emphasizes the importance of minimizing the length of hospitalisation whenever possible.

Insertion of a nasogastric tube was also positively associated with having an ESBL-E infection or carriage during the outbreak. As a procedure, nasogastric tubing is rather invasive, since the reusable tube is passed from nostril to stomach and can thus cause mucosal trauma in the upper airways. These factors may contribute to the direct transmission of environmental pathogens. No ESBL-E was found in the actual nasogastric tubes or in the lubricant in our study, but we found ESBL-E in stomach drench pumps. However, these isolates differed from those originating from horses. The transmission of ESBL-E from the environment or equipment onto the muzzle and into the nostrils may enhance the spread of these organisms to the gastrointestinal tract during procedures such as nasogastric tubing or dental care.

In human studies, numerous species of *Enterobacterales* were found in the lumen of enteral feeding tubes already after a few hours [51]. As many ESBL-E can form biofilms, proper cleaning and disinfection of the equipment are important to prevent nosocomial transmission. Tubes can be washed in a disinfecting washer, or they can be soaked in detergent containing fat and protein-dissolving agents, manually scrubbed, and finally disinfected. As a result of this finding by our study, the standard cleaning procedure for nasogastric tubes and stomach drench pumps was enhanced.

Further, mare–foal pairs demonstrated a higher risk for being positive for ESBL-E. In humans, pregnant women that are faecal carriers of ESBL-E are more likely to transmit ESBL-E to their new-born infants than mothers with no ESBL-E [52]. Digestive tract colonization with ESBL-E in humans also increases the risk of acquiring an ESBL-E infection during hospital care [53]. Foals often receive intense care and thus frequent treatments, including antimicrobials. They are housed in a stall together with their mare and are in close contact with both the mare and the hospital environment. A study by Dolejska et al. showed that the antimicrobial treatment of the foal resulted in excretion of ESBL-producing *E. coli* also by the mare and the horses from the neighbouring stalls, as well as contamination of the stable environment [37]. A study by Damborg et al. also showed that horses negative for ESBL-E on admission were all found positive for faecal carriage of ESBL-E after receiving antimicrobial therapy during hospital care [54].

Schoster et al. [55] showed that antimicrobial treatment of a hospitalised horse was a risk factor for acquiring ESBL-E. In our study, antimicrobial treatment was associated with being positive for ESBL-E in the univariable analysis, but not in the multivariable analysis. One explanation might be that hospitalised horses often receive antimicrobial treatment parenterally, which prolongs the length of stay. The length of hospitalisation was shown to be a risk factor for being positive for ESBL-E. Association with the length of stay may be the reason for antimicrobial treatment alone not having been stated as a risk factor in the multivariable analysis.

As the study was retrospective, we were unable to show causality behind correlation. We also had to collect data from the patients in the medical reports, and therefore there may have been differences between the veterinary staff of the hospital regarding what information they recorded for each patient. This might have affected the values for some variables we set for each horse.

Another limitation of the study was that the incoming patients were screened for ESBL-E after spending > 24 h in the hospital, and not immediately on admission. Therefore, it is possible that some horses might have been carriers already before entering the hospital. Humans that are ESBL-E carriers on admission to hospital are at higher risk of developing an infection caused by an ESBL-E [56]. A similar association has been suggested in horses, but evidence that faecal carriage of ESBL-E would correlate with a higher incidence of infection, is lacking thus far [34]. However, strict infection surveillance was already in place before the *K. pneumoniae* outbreak occurred at the EVTH. All infection sites were required to be cultured immediately and before institution of an antimicrobial therapy. This allowed for any infection caused by an ESBL-E to be detected rapidly. From the standpoint of epidemiology and disease prevention, a cross-sectional study on local prevalence is suggested.

## Conclusions

*Klebsiella pneumoniae* ST307:CTX-M-1 and other ESBL-E are capable of giving rise to an outbreak in equine hospital settings, which highlights the importance of epidemiological surveillance of ESBL-producing *Enterobacterales* in veterinary practises. A broad heterogeneity of ESBL-E species and similar ESBL genes among the species were revealed in hospitalised horses, which may indicate horizontal gene transfer. It is therefore important to practise sufficient hygiene measures in handling the patients and cleaning the equipment, and also to maintain infection surveillance to prevent nosocomial transmission of resistant pathogens. Equine practices should also try to minimize the length of hospitalisation as it may reduce the risk for the spread of ESBL-E in equine patients.

## Supplementary Information

**Additional file 1:** Equine patient data collection for ESBL-E risk factor analysis. List of patient information, including variables and comments, used in the data collection of the study.

## Data Availability

The datasets used and/or analysed during the current study are available from the corresponding author on reasonable request.
