# Supplementary material for: Characterisation of and risk factors for extended-spectrum β-lactamase producing Enterobacterales (ESBL-E) in an equine hospital with a special reference to an outbreak caused by Klebsiella pneumoniae ST307:CTX-M-1
Source: Acta Vet Scand. 2022 Feb 9;64:4. doi: 10.1186/s13028-022-00621-6 (PMC8827190; doi:10.1186/s13028-022-00621-6)
Supplement: Supplementary file 1 — Additional file 1: Equine patient data collection for ESBL-E risk factor analysis. List of patient information, including variables and comments, used in the data collection of the study. [file 13028_2022_621_MOESM1_ESM.pdf]

Additional file 1. Equine Patient Data Collection for ESBL-E Risk Analysis

Equine patient data:

- Patient number
- Name of the horse
- Gender: not known / mare / stallion / gelding
- Breed: not known / breed
- Date of birth
- Date of arrival to the Equine Veterinary Teaching Hospital (EVTH): the same visit to the EVTH when the first ESBL positive or the last ESBL negative specimen was taken
- Age in years: age in years on the day of admission to the EVTH
- Type of case: individual horse / mare–foal pair
- Information on whom, mare or foal, needed the treatment during the EVTH visit
- ESBL-positive specimen: yes / no
- Bacterial species detected in the ESBL positive specimen
- ESBL-positive specimen type: infection / screening / both
- Specification of the infection site
- ESBL-negative screening result during the same treatment period of the horse: yes / no
- The date of the first ESBL positive or the last negative specimen
- The diary number of the first ESBL positive or the last negative specimen
- Length of the hospital stay at the EVTH: marked in days before the sampling mentioned above
- Previous visits at the EVTH approximately one month prior to the outbreak (March 9th, 2013) and the case-control treatment period: yes / no
- Number of previous treatment days

- Patient treated in other equine hospital during 03 – 09/2013 and the case-control treatment period: yes / no
- Previous visit abroad during the last 3 months: yes / no; if yes, to which country

<u>Treatment period:</u>

- Classification of the urgency during the case-control period: outpatient / emergency service
- Passing of a nasogastric tube during the case-control period: yes / no
- Number of nasogastric tubing procedures
- Number of nasogastric tubing procedures per day: once a day / twice or three times a day / four times or more a day
- Number of days carrying out nasogastric tubing procedures
- Observations on the nasogastric tubing procedure: comments
- Surgical procedure requiring anesthesia or penetration of skin/mucous membrane at the EVTH during the case-control period: yes / no
- Type and name of the surgical procedure
- Systemic antimicrobial treatment during the case-control period: yes / no
- Number of days of the systemic antimicrobial treatment
- Treatment with penicillin / ceftiofur / gentamicin / doxycycline / sulfonamide-trimethoprim / enrofloxacin / metronidazole: yes / no per antimicrobial agent
- Duration of the antimicrobial treatment (antimicrobial agents mentioned above) in days
- Other antimicrobial treatment: yes / no
- Specification of other antimicrobial agents (for example polymyxin B, rifampicin, clarithromycin) and duration in days

<u>Notes:</u>

- Observations during the data collection
